# Supplementary material for: MMM and MMMSynth: Clustering of heterogeneous tabular data, and synthetic data generation
Source: PLoS One. 2024 Apr 17;19(4):e0302271. doi: 10.1371/journal.pone.0302271 (PMC11023594; doi:10.1371/journal.pone.0302271)
Supplement: S1 File — (PDF) [file pone.0302271.s001.pdf]

Supplementary information
MMM and MMMSynth: Clustering of heterogeneous tabular data, and synthetic data generation

Chandrani Kumari and Rahul Siddharthan

This supplement explores other clustering metrics than Adjusted Rand Index (used in main paper), corresponding to figure 2 of that paper.

The last two measures (Davies-Bouldin and Silhouette) are intrinsic measures that require a distance metric between rows, for which we used Euclidean distance here; however, the MMM algorithm does not use Euclidean distance but likelihood based on an underlying unknown probability distribution. Therefore they are to be interpreted with caution but are included for completeness.

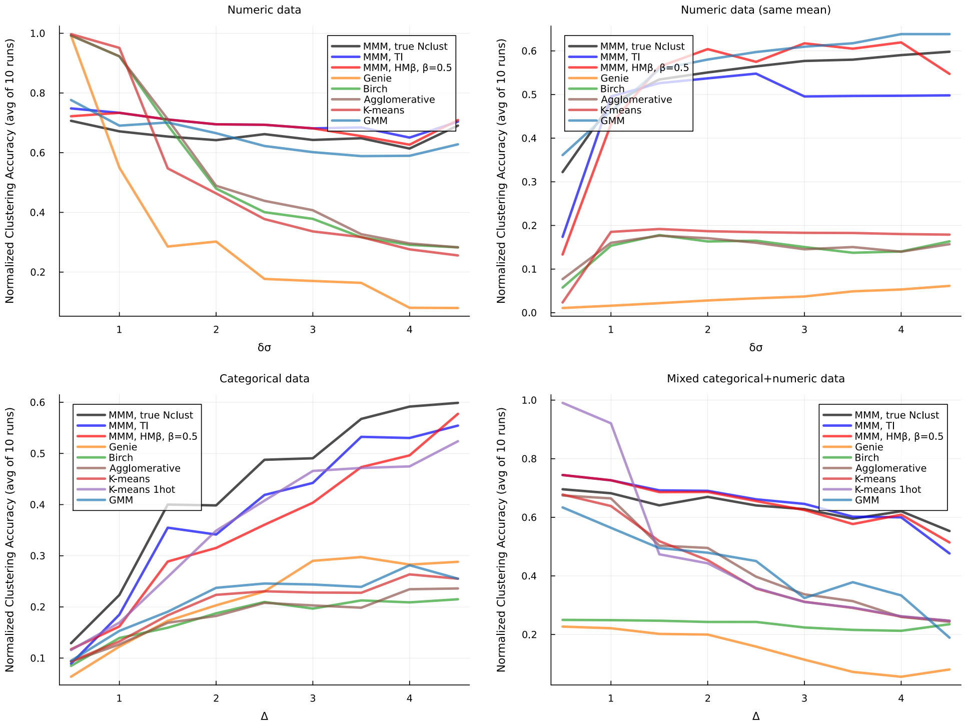

Figure 1: Normalized Clustering Accuracy

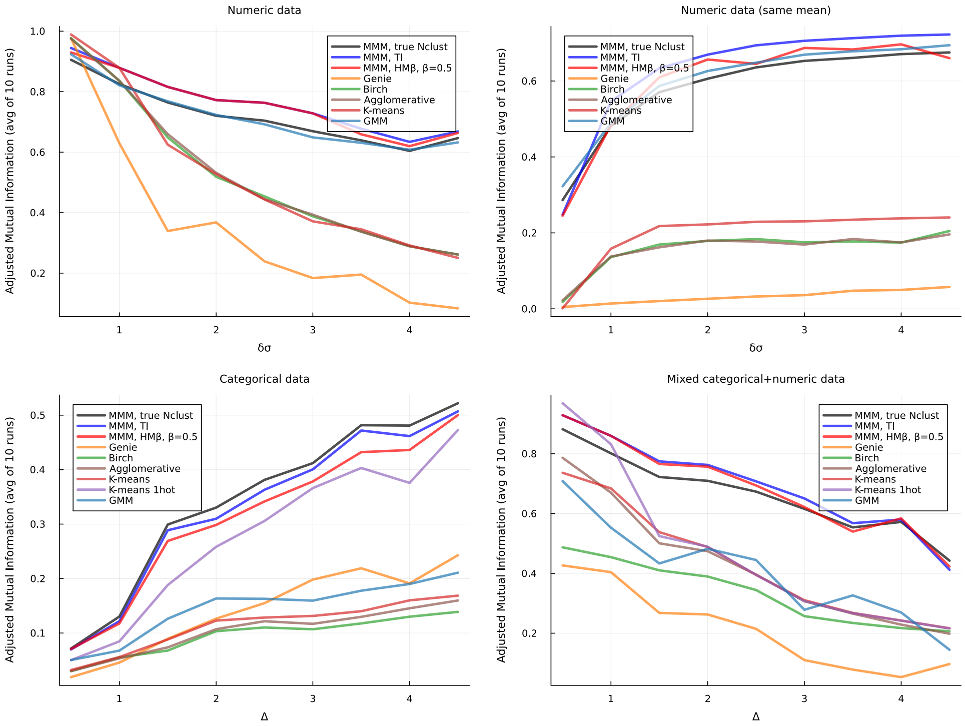

Figure 2: Adjusted Mutual Information

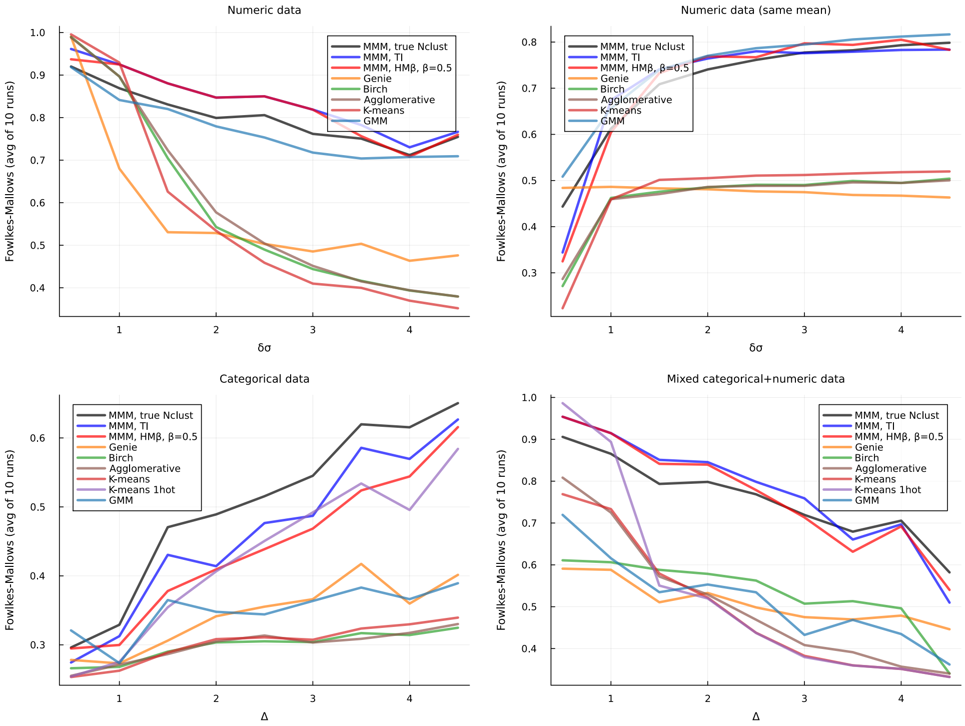

Figure 3: Fowlkes-Mallows score

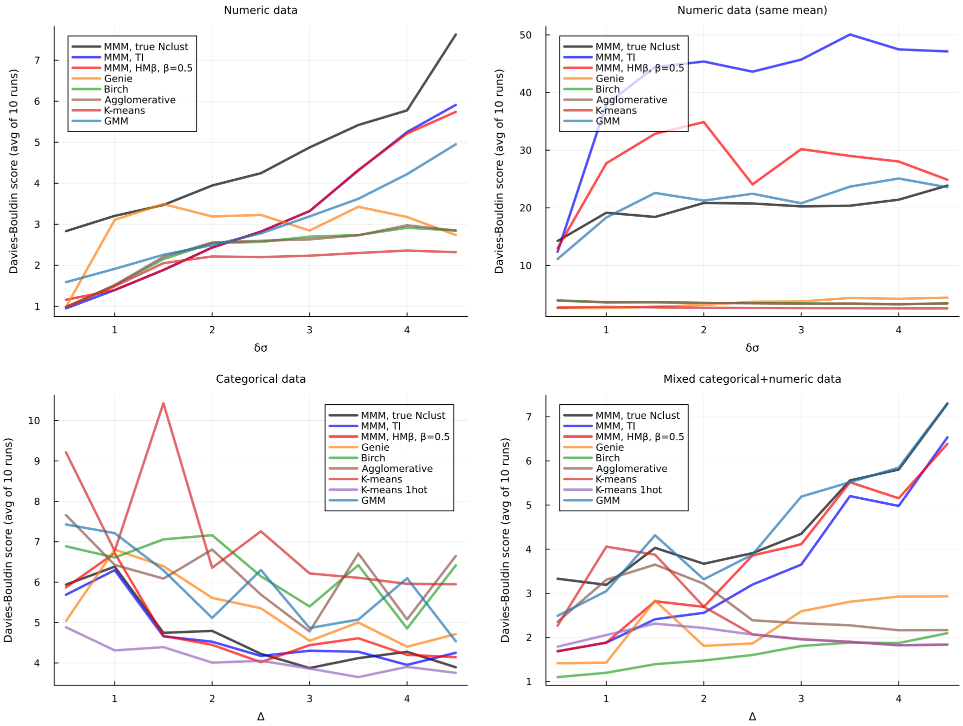

Figure 4: Davies-Bouldin score

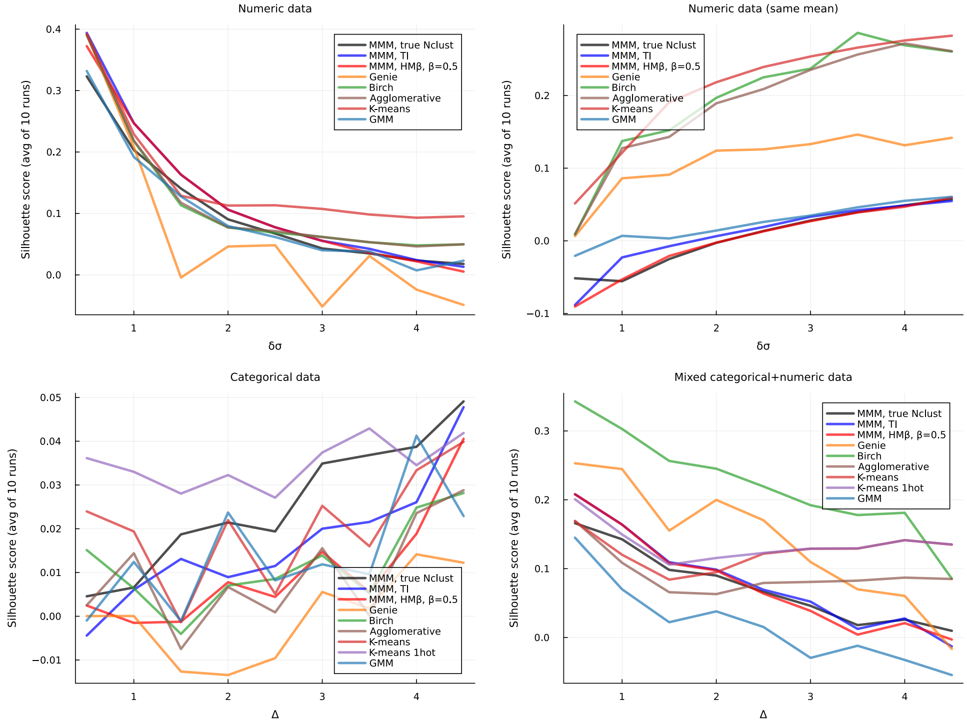

Figure 5: Silhouette score
