# Supplementary material for: MMM and MMMSynth: Clustering of heterogeneous tabular data, and synthetic data generation
Source: PLoS One. 2024 Apr 17;19(4):e0302271. doi: 10.1371/journal.pone.0302271 (PMC11023594; doi:10.1371/journal.pone.0302271)
Supplement: Attachment — Submitted filename: Reviewer comments.docx [file pone.0302271.s002.docx]

The present manuscript by Kumari and co-authors introduces a new clustering method (named MMM) and a new method for the generation of synthetic data (named MMMSynth). The manuscript presents the mathematical formulations for their methods introducing the reader to the underlying concept of the proposed method. The performance (from the perspective of two metrics) of the proposed clustering method has been evaluated against other five clustering algorithms on three synthetically generated datasets with varying values of standard deviations (decreasing and increasing the difficulty of clustering). Moreover, the authors have validated their method (in three variants, with two approximations for the number of clusters and the true number of clusters) is in comparison to five other clustering algorithms (that have been given the true number of clusters) on multiple datasets that have binary (seven datasets) and multiclass (eight datasets) labels from the perspective of a performance metric.

# Major points:

Section 3.1.1: As you mention, the increase of the δσ on the x-axis will result in more similar clusters, nevertheless, all methods are becoming worse for Figure 2 panels A and D with higher values of δσ (that indicate more similar clusters). This is not discussed in the manuscript. The performance metric choice changing across different data sets (Figure 2 panel A vs panels B,C,D) is again not discussed. I would recommend either keeping ARI across all as the normalized accuracy appears just in Figure 2A or extending all analyses to include both metrics.

Section 2.6: Multiple perspectives are offered from multiple metrics, such as Adjusted Mutual Information/V-Measure, Purity, Fowlkes-Mallows. And even from internal metrics such as Silhouette, Davies-Bouldin, Calinski-Harabasz. This section has two sentences at this moment, I would recommend extending and discussing the choice of metrics as the normalized accuracy is used in a single analysis.

Overall, the figures are hardly discussed/interpreted in the manuscript, they are only referenced. I would recommend extending the manuscript with an interpretation of the results. Figures 2 and 3 could be improved, as the same methods are used, you could have the same legends (the addition of sklearn in the names of Figure 3 is not relevant as they are exactly the same methods as in figure 2). From Figure 2 to Figure 3, the name "MMM, true nClust" has been changed into "MMM (fixed)", making the manuscript harder to read. As a question, why is Figure 3 lacking the MMM, HMβ option? This is not discussed either. Figure 3 could have “ARI” added as the x-axis label.

Regarding the claims in section 4. Discussion, that the proposed methods outperform others:

- In Figure 3 panel A, GMM actually shows a better performance than the proposed method (even when the proposed method is given the true number of clusters) for all datasets.
- The results from Figure 4 do not exactly show this. TVAE might even outperform the proposed method on average for RandomForest and GC is a close contender as well. By averaging the performances from Figure 4, panel A, it seems that TVAE has overall a higher value. For panel B, the proposed method will probably have a slightly higher value but not by much, I would estimate around 3%. I would recommend more analyses, as at this point, the proposed generation method does not seem to bring a considerable improvement in comparison to TVAE.

# Minor points:

Section 3.1: As far as I understand δσ will result in more dispersed clusters as it changes the standard deviation of the cluster. Thus, by similar do you mean that they will have more similar densities?

Section 2.8: You specify that the first list of datasets have binary output variables. But the abalone dataset has an integer value in the number of rings which is not binary. At least that is the case of the abalone dataset shown in the link.

Section 2.3: The mathematical formulations seem sound, although I have a question, shouldn’t it be μ_n_ in equation (5)?

Section 2.3: In the first paragraph, there is a period added after reference [13], while the sentence continues after it.

Section 2.7.1: In the second paragraph, “available” written as “availbale”.

Section 4: The GC acronym has been defined above, yet it is not used in the 4. Discussion section

I would recommend extending the github code. It is hard to use with the only output available as a file of labels. A plot (2D/3D through PCA) of the result of clustering a synthetically generated dataset in comparison to another clustering method (such as K-Means), would be a helpful addition. At this point, validation of the code requires additional code to be written by others.
